# Supplementary material for: RNA Sequencing-Based Identification of Ganglioside GD2-Positive Cancer Phenotype
Source: Biomedicines. 2020 May 30;8(6):142. doi: 10.3390/biomedicines8060142 (PMC7344710; doi:10.3390/biomedicines8060142)
Supplement: Supplementary file 1 [file biomedicines-08-00142-s001.zip › Supplementary2.docx]

**Abbreviations**

LacCer synthase - B4GALT6 (Beta-1,4-galactosyltransferase 6);

GM3 synthase - ST3GAL5 (ST3 Beta-Galactoside Alpha-2,3-Sialyltransferase 5);

GD3 synthase - ST8SIA1 (ST8 Alpha-N-Acetyl-Neuraminide Alpha-2,8-Sialyltransferase 1);

GT3 synthase - ST8SIA5 (ST8 Alpha-N-Acetyl-Neuraminide Alpha-2,8-Sialyltransferase 5);

GD2 synthase (GM2/GD2 synthase) - B4GALNT1 (Beta-1,4-N-Acetyl-Galactosaminyltransferase 1);

GD1b synthase (GM1a/GD1b synthase) - B3GALT4 (Beta-1,3-Galactosyltransferase 4);

Cer - Ceramide, N-Acylsphingosine;

GlcCer - Glcβ1Cer;

LacCer - Galβ1Cer;

GA1 - Galβ1,3GalNAcβ1,4Galβ1,4Glcβ1Cer;

GA2 - GalNAcβ1,4Galβ1,4Glcβ1Cer;

GM3 - Neu5Acα2,3Galβ1,4Glcβ1Cer;

GM2 - GalNAcβ1,4(Neu5Acα2,3)Galβ1,4Glcβ1Cer;

GM1a - Galβ1,3GalNAcβ1,4(Neu5Acα2,3)Galβ1,4Glcβ1Cer;

GD3 - Neu5Acα2,8Neu5Acα2,3Galβ1,4Glcβ1Cer;

GD2 - GalNAcβ1,4(Neu5Acα2,8Neu5Acα2,3)Galβ1,4Glcβ1Cer;

GD1b - Galβ1,3GalNAcβ1,4(Neu5Acα2,8Neu5Acα2,3)Galβ1,4Glcβ1Cer;

GT3 - Neu5Acα2,8Neu5Acα2,8Neu5Acα2,3Galβ1,4Glcβ1Cer;

GT2 - GalNAcβ1,4(Neu5Acα2,8Neu5Acα2,8Neu5Acα2,3)Galβ1,4Glcβ1Cer;

GT1c - Galβ1,3GalNAcβ1,4(Neu5Acα2,8Neu5Acα2,8Neu5Acα2,3)Galβ1,4Glcβ1Cer;
Neu5Ac - N-Acetyl-neuraminic acid;

Glc - Glucose;

Gal - Galactose;

GalNAc - N-Acetyl-galactosamine.
