# Supplementary figures and images for: RNA Sequencing-Based Identification of Ganglioside GD2-Positive Cancer Phenotype
Source: Biomedicines. 2020 May 30;8(6):142. doi: 10.3390/biomedicines8060142 (PMC7344710; doi:10.3390/biomedicines8060142)

Log<sub>10</sub>(sum of gene expression)

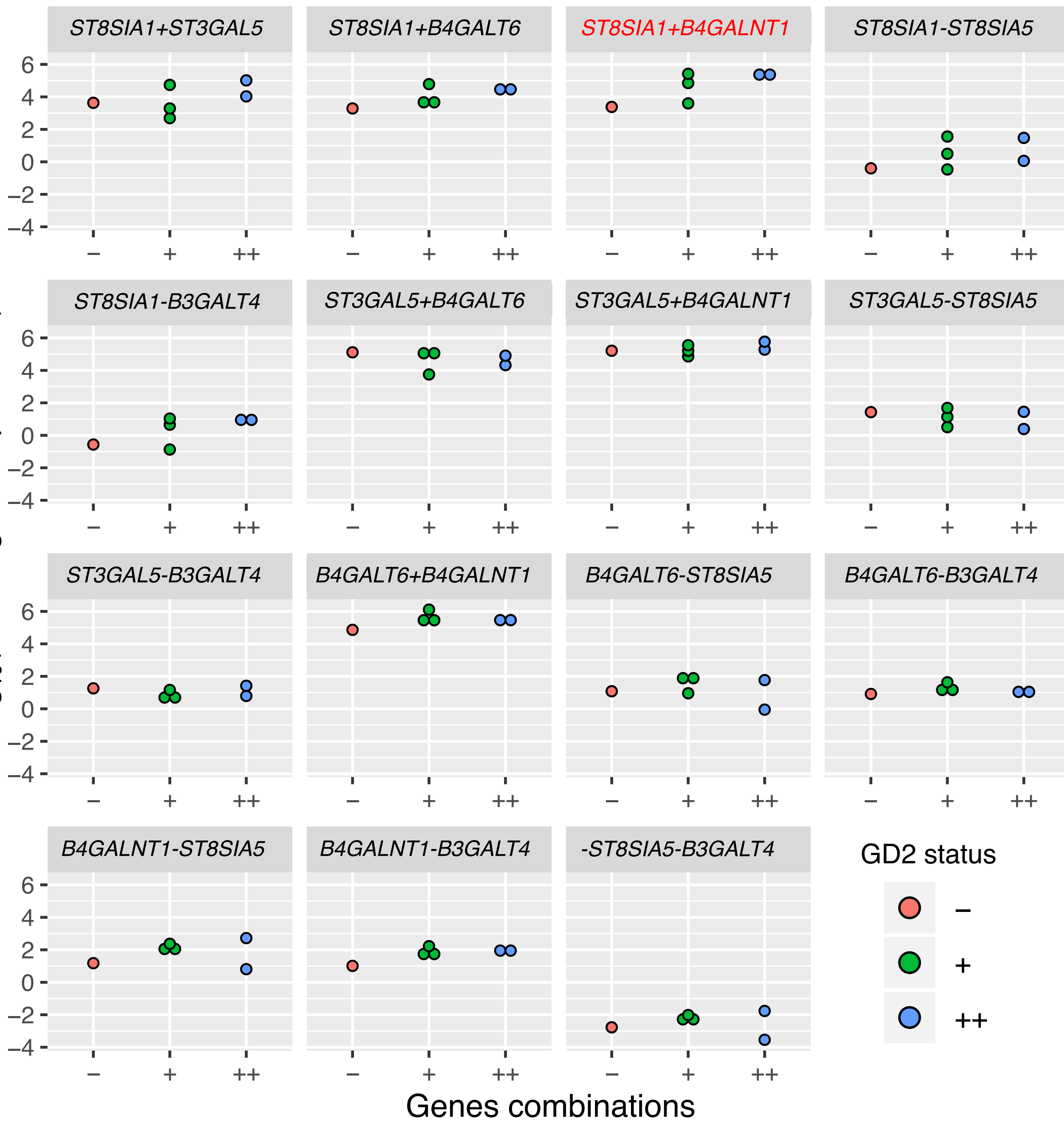

Supplement: Supplementary file 1 [file biomedicines-08-00142-s001.zip › Figure_S1.pdf]
